# Determinants of rabies knowledge, attitudes, and practices among secondary and vocational school students in Maha Sarakham Province, Thailand

**DOI:** 10.1186/s12917-026-05796-y

**Published:** 2026-08-03

**Authors:** Kanokwan Suwannarong, Kannika Thammasutti, Satitpong Promsatit, Alongkorn Amonsin, Claire Lajaunie, Natapol Pumipuntu

**Affiliations:** 1 Center of Excellence for Emerging and Re-emerging Infectious Diseases in Animals, Faculty of Veterinary Science, Chulalongkorn University, Bangkok, Thailand; 2 SUPA71 Co., Ltd, Bangkok, Thailand; 3 Department of Livestock Development, Amnat Charoen Provincial Livestock Office, Amnat Charoen, Thailand; 4 French National Institute of Public Health and Medical Research (INSERM), Based at LPED Research Unit, Marseille, France; 5 IRL HealthDEEP, CNRS-Kasetsart University-Mahidol University, Bangkok, Thailand; 6 One Health Research Unit, Mahasarakham University, Maha Sarakham, Thailand; 7 Faculty of Veterinary Sciences, Mahasarakham University, Maha Sarakham, 44000 Thailand

**Keywords:** Attitude, Knowledge, KAP, Practice, Rabies, Student, Thailand

## Abstract

Rabies is a critical zoonotic disease that is found throughout the world, particularly in Africa, Asia, and Latin America. Once a clinical sign appears, the fatality rate rises to 100% globally. In Thailand, children under the age of 15 had the highest annual reported exposure rate to rabies infection. One of the most significant barriers to disease prevention and control is a lack of public awareness about rabies, particularly among children and young people. The study’s goal was to determine knowledge, attitude, and preventive practice (KAP) about rabies and associated factors among secondary and vocational students in Maha Sarakham, Thailand, in 2023. An analytic cross-sectional survey was carried out from January to May 2023, with a total of 628 study participants. Data was collected through online and on-site questionnaires. Descriptive analysis was carried out. Inferential statistics analysis was also performed to identify factors associated with rabies KAP among the target participants.

Most participants demonstrated a moderate level of knowledge (55.89%), neutral attitudes (81.37%), and moderate preventive practices (72.77%) toward rabies. Students who had experience raising animals, had previously studied zoonotic diseases, had a history of animal bites or scratches, or had received rabies vaccination showed significantly higher knowledge and practice scores (*p* < 0.05). Knowledge and attitudes were positively correlated with preventive practices (*r* = 0.14 and *r* = 0.28, respectively; *p* < 0.05). The findings suggest a significant need for rabies awareness programs targeting school and college students within the community. The materials designed to promote KAP regarding rabies should address specific topics, including the risk of rabies, modes of transmission, the significance of first aid, health-seeking behavior following dog bites or cat scratches, and preventive measures for both pets and community dogs and cats.

**Supplementary Information:**

The online version contains supplementary material available at https://doi.org/10.1186/s12917-026-05796-y.

## Introduction

Rabies is considered one of the infectious diseases that affects all mammals [1]. A Lyssavirus of the family *Rhabdoviridae* causes rabies and is usually transmitted to humans through the bite of a rabid animal [2]. Although rabies remains a fatal viral infection once clinical symptoms appear, it can be effectively prevented through vaccination, either before or after exposure [3]. Despite the availability of effective preventive measures, the disease continues to cause considerable mortality in both humans and animals, especially in regions across Asia and Africa [4–6]. The significant incidence of rabies-related fatalities in a number of rabies-endemic countries, such as Pakistan, indicates the presence of inadequate human and animal rabies prevention and control initiatives [2, 7]. Domestic dogs are the principal reservoir and source of urban rabies, accounting for approximately 99% of human rabies cases worldwide [3]. In many rabies-endemic countries, transmission within dog populations remains the primary source of human infection, making dog vaccination, responsible pet ownership, and public awareness essential components of rabies prevention and control [3, 4]. Nevertheless, outbreaks continue to occur in some rabies-endemic urban areas where control measures are inadequate or inconsistently implemented [8–10].

In Thailand, rabies surveillance by the Bureau of Epidemiology, Department of Disease Control, reported a total of 20 confirmed human rabies cases between 2020 and 2024, with a median of four cases per year [8]. In 2024, four confirmed human rabies deaths were reported (case fatality rate 100.0%), corresponding to an incidence rate of 0.01 per 100,000 population [8]. As of March 2025, four additional confirmed cases were reported, all of whom died (case fatality rate 100.0%) [8]. The median age of cases in 2024 was 52 years, while in early 2025 it was 27 years [8]. The most common individual risk factor identified was that some individuals exposed to potentially rabid animals did not receive timely and complete post-exposure prophylaxis (PEP). Improving the management of exposed patients and ensuring prompt access to PEP remain essential components of human rabies prevention and control [8]. Therefore, the Thai government has reinforced rabies prevention and control programs through collaboration between health authorities, livestock departments, and local communities [9]. Effective rabies control requires both institutional commitment and active community participation [3]. Enhancing public awareness of vaccination, responsible pet ownership, and timely post-exposure treatment remains essential, particularly in provinces where rabies persists as an endemic threat [3]. Interestingly, children are a population group at high risk of rabies exposure in Thailand. Approximately one-third of Thai children are bitten by dogs at least once by the age of 15 [10].

Potential risk assessment related to rabies infection and the level of prevention can be measured from several KAP surveys conducted in various communities, and the findings could be applied to rabies-endemic areas by guiding targeted health education campaigns, improving rabies vaccination coverage among dogs, and informing policy decisions to strengthen community-based rabies prevention and control programs [11–13]. The lessons learned from these efforts could motivate communities in rabies-endemic areas to prevent the disease and guide the development of culturally appropriate intervention programs. This analytic cross-sectional study aimed to assess the knowledge, attitudes, and practices (KAP) regarding rabies control and prevention among secondary and vocational students in Maha Sarakham, Thailand. This province was chosen because it consists of numerous academic institutions and student populations, which facilitates the generation of baseline data. The findings of this study are expected to be crucial in identifying significant gaps in knowledge, awareness, and practices concerning rabies, thereby informing future rabies control and prevention strategies in this province and beyond.

## Materials and methods

### Study design and study period

An analytical cross-sectional survey was carried out using both on-site and online data collection methods from January to May 2023. A face-to-face, paper-based questionnaire was utilized to gather data on-site, while a Google Form was developed to collect data online from the target students.

### Study locations and settings

**Fig. 1:**
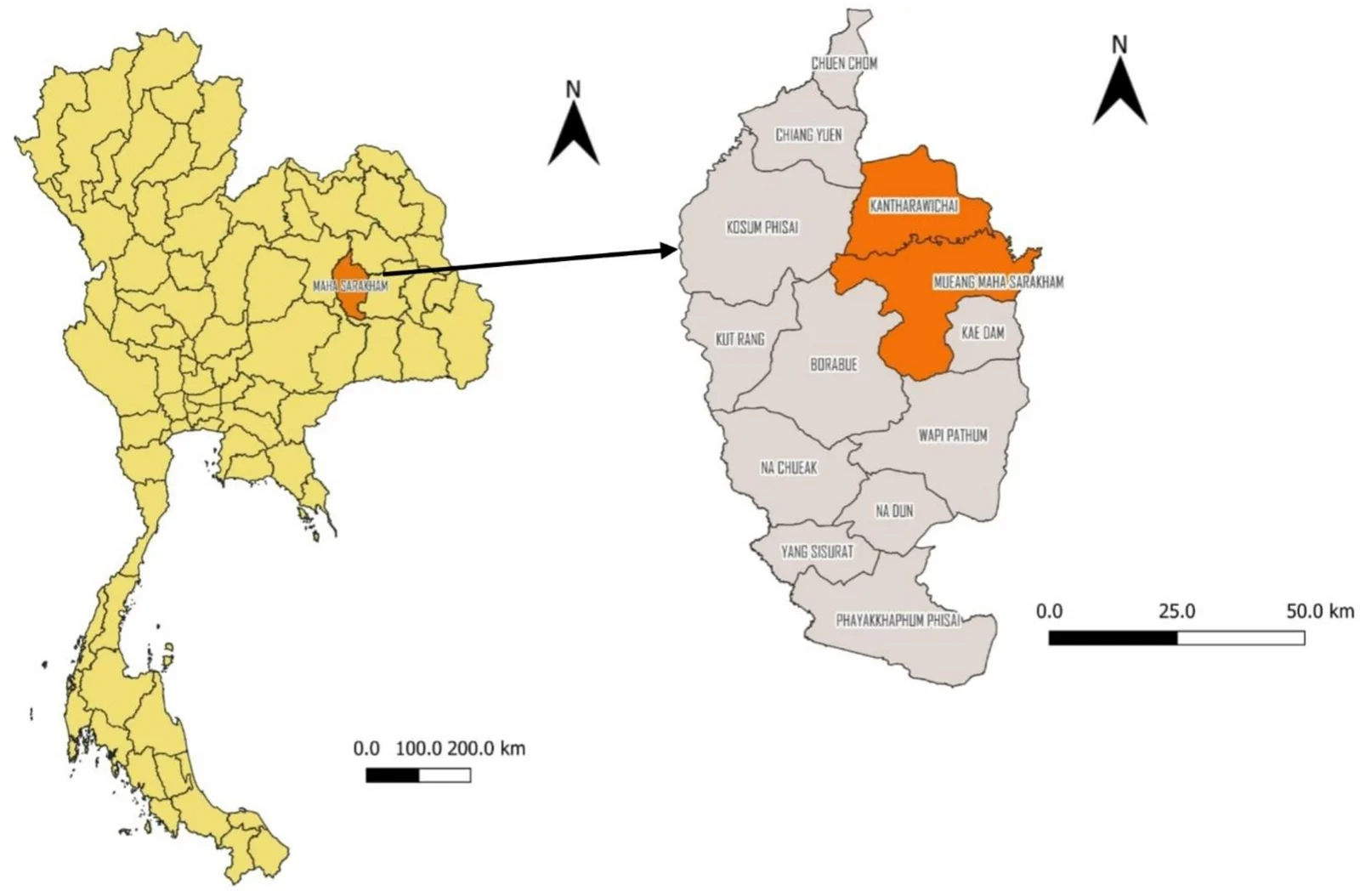
Map of the study locations in Maha Sarakham Province, Thailand, including two districts (Muang Maha Sarakham and Kantarawichai). Remarks: The map was generated using Quantum GIS version 3.4.5 (QGIS Development Team, 2019)

Maha Sarakham is a northeastern province in Thailand. The neighboring provinces are Kalasin, Roi Et, Surin, Buriram, and Khon Kaen. It comprises 13 districts and 133 subdistricts, with an approximate population of 900,000–1,000,000 people. (Fig. 1) This province was selected for this study since it has several academic institutions and student populations that might be able to obtain baseline information for the KAP levels among the students. Two districts of one province (Muang Maha Sarakahm and Kantarawichai) were selected for this study. The two study locations include several high schools and vocational institutions (Fig. 1), such as Sarakhampittayakhom School, Rajabhat Maha Sarakham University Demonstration School, Phadungnaree School, Mahasarakham Technical College, Mahasarakham Vocational College, Mahasarakham University Demonstration School (Secondary), and Tha Khon Yang Pittayakhom School.

### Sample size estimation, study participants, and sampling methods

The sample size was estimated using Cochran’s formula for estimating a population proportion in a large population [14].

$$n=\frac{{Z}^{2}\times\:p\times\:\left(1-p\right)}{{e}^{2}}$$

where *n* is the required sample size, *Z* is the standard normal deviate corresponding to the desired confidence level, *p* is the estimated proportion of the population with the characteristic of interest, and *e* is the acceptable margin of error. Since no previous studies on rabies-related knowledge among the target population were available, a conservative prevalence estimate of 50% (*p* = 0.50) was assumed. Using a 95% confidence level (*Z* = 1.96) and a margin of error of 4% (*e* = 0.04), the minimum required sample size was calculated to be 601 participants. To improve the precision of the estimates and ensure an adequate sample size, a total of 628 students were ultimately included in the study.

The target participants were youth aged 12–17 years who had been studying in one of the academic institutions within the study areas for at least three months and who voluntarily participated in the study. This study employed both on-site surveys and online questionnaires for data collection.

A multistage sampling approach was employed, involving the purposive selection of study sites and educational institutions, followed by simple random sampling of eligible students for the on-site survey and voluntary participation through an online questionnaire. Details of the sampling stages are as follows:

- Stage 1 included the simple random selection of two districts, including Muang Maha Sarakham and Kantarawichai, from the 13 districts of Maha Sarakham Province. These districts were represented in urban, peri-urban, and rural settings within the province.
- Stage 2 consisted of the selection of seven educational institutions within the chosen districts, including 5 high schools and 2 vocational institutions.
- Finally, Stage 3 consisted of simple random sampling of eligible students within each selected institution for participation in the on-site survey, supplemented by voluntary participation through an online questionnaire.

Students were eligible to participate if they were currently enrolled in one of the selected high schools and vocational institutions in Maha Sarakham province during 2023 and voluntarily agreed to join either the on-site or online survey. Exclusion criteria were applied to students who were not officially registered in the study area at the time of data collection, those younger than 12 years or older than 17 years, students who declined to provide informed consent or whose parents/guardians did not provide consent for minors, and individuals unable to complete the questionnaire due to language barriers, learning disabilities, or other communication difficulties. Discontinuation criteria included students who chose to withdraw from participation at any point and questionnaires that were not fully completed.

All procedures involving human participants were approved by the Mahasarakham University Ethics Committee for Research Involving Human Subjects (Approval Number: 238/2022) and conducted in accordance with the ethical guidelines of the National Research Council of Thailand (NRCT). Although participants aged 12–17 years are considered minors, individuals in this age group are regarded as capable of understanding research information under national ethical guidelines. Therefore, a combined informed consent form was used for both participants and their parents or legal guardians, requiring signatures from both parties to ensure that parental consent and participant assent were obtained simultaneously.

### Data collection tool and implementation

The data was collected using both on-site surveys and online questionnaires via Google Forms. The structured questionnaire in Thai was adapted based on a previous study by Kiratitana-Olan et al. (2021) [15] and further developed following a review of relevant literature on rabies in Thailand [16–21]. The questionnaire was used to determine potential differences associated with the data collection mode, sociodemographic characteristics of respondents from the on-site and online surveys were compared. Variables including age, gender, and educational level were assessed prior to combining the datasets for analysis. The questionnaire was refined following pilot testing with 10 individuals who were not included in the study sample and consisted of 40 multiple-choice items. The survey began with questions on demographic characteristics, including gender, age, educational level, and prior experience related to zoonotic diseases and rabies. Following the three sections, including (1) the knowledge section (15 items) assessed basic understanding of rabies and key preventive measures in both humans and animals. One point was scored for each correct response and zero points for an incorrect response, resulting in a raw score ranging from 0 to 15. Raw scores were subsequently converted to a standardized 50-point scale and classified as poor (0.00–16.67), fair/ moderate (16.68–33.33), or good (33.34–50.00); (2) The attitude section (10 items) explored perceptions of rabies severity and views on prevention. The attitude responses were scored using a 5-point Likert scale ranging from 1 (strongly disagree) to 5 (strongly agree). The mean attitude score was calculated and categorized as negative (1.00–2.33), neutral (2.34–3.67), or positive (3.68–5.00) (3) The practice section (15 items) was evaluated for behaviors and preventive actions associated with rabies exposure activities using a 4-point frequency scale. Practice scores were converted to a standardized 60-point scale and categorized as unsatisfactory (0.00–20.00), average/moderate (21.00–40.00), or good (41.00–60.00). The detailed questionnaire items, response options, and scoring criteria are provided in Supplementary Table 1.

### Data analysis

The data were reviewed, verified, and managed daily by the study team prior to analysis. All statistical analyses were performed using IBM SPSS Statistics for Windows, version 26.0 (IBM, Armonk, NY, USA).

During the analysis steps, we compared the sociodemographic characteristics of respondents from face-to-face interviews and online questionnaires and found no statistically significant differences in key variables, including age, gender, and educational level (*p* > 0.05). Therefore, data from both groups were combined for analysis. In addition, continuous variables were assessed for normality using the Shapiro–Wilk test. As the KAP scores did not meet the assumption of normal distribution (*p* < 0.05), non-parametric statistical methods were applied. Skewed data were summarized as median and interquartile range (IQR), whereas categorical variables were presented as frequencies, percentages, and 95% confidence intervals (CI).

The cumulative score obtained for questions based on the three response criteria (knowledge, attitudes, and practices towards rabies, respectively) was assessed. Dividing into three groups by class interval involved creating categories based on the distribution of quantiles to represent poor, fair, and good levels. The impact of sociodemographic variables (independent variables) was explored as a predictor of KAP (dependent variables). To describe each dependent variable that met non-normal distribution assumptions, a Mann-Whitney U test was used to compare between two groups and a Kruskal-Wallis rank test to compare across three or more groups. When the Kruskal–Wallis rank test yielded a significant result, pairwise comparisons were performed using the Mann–Whitney U test to identify specific groups with statistically significant differences. Finally, Spearman’s correlation coefficient was used to assess the strength and direction of the monotonic relationship between the dependent variables based on their ranked data. Correlation coefficients close to − 1 or + 1 were interpreted as indicating a strong monotonic relationship, whereas values closer to 0 were interpreted as indicating a weak relationship [22, 23].

In addition, bivariable analyses were performed to examine the associations between participant characteristics and good knowledge, positive attitudes, and good practices regarding rabies prevention. Pearson’s chi-square test was used for categorical variables, whereas Fisher’s exact test was applied when the expected cell count was less than five. Variables with a *P*-value < 0.20 in the bivariable analyses were entered into separate multivariable logistic regression models for knowledge, attitudes, and practices. A backward stepwise elimination procedure was applied to obtain the final models. Adjusted odds ratios (AORs) with 95% confidence intervals (CIs) were calculated to identify independent factors associated with each outcome. Statistical significance was defined as a two-sided *P*-value < 0.05.

## Results

### Profiles of the study participants

Six hundred twenty-eight participants participated in this study, which consisted of 397 (63.22%) females, 191 (30.41%) males, and 40 (6.37%) non-binary. Most of them were aged over 15 years (292, 46.50%), followed by 15 years (39.01%), and under 15 years (14.49%), respectively. They studied in the science-mathematics program of high school (144, 22.93%), followed by the art program of the high school (122, 19.43%), junior high school (91, 14.79%), and vocational college (8, 1.27%). Most participants lived in rural areas (289, 46.02%), followed by urban areas (181, 28.82%) and peri-urban areas (158, 25.16%). Regarding animal ownership, 290 participants (46.18%) reported raising companion animals only, 86 (13.69%) reported raising both livestock and companion animals, and 65 (10.35%) reported raising livestock only. In contrast, 187 participants (29.78%) reported not raising any animals (Table 1).

**Table 1:** Socio-demographic characteristics of the study participants from secondary schools and vocational institutions in Maha Sarakham Province, Thailand (*n* = 628)

Variables	Descriptions	Number (Percent)	Reliability (95%)	
			Lowest	Highest
Gender	Male	191 (30.41)	26.93	34.13
	Female	397 (63.22)	59.36	66.91
	Others	40 (6.37)	4.7	8.57
Age-group	Under 15 years	91 (14.49)	11.94	17.47
	15 years of age	245 (39.01)	35.26	42.90
	Over 15 years	292 (46.50)	42.62	50.42
Educational attainment levels	Junior high school	354 (56.37)	52.45	60.21
	Vocational college	8 (1.27)	0.64	2.53
	High school, Arts program, and other programs	122 (19.43)	16.51	22.72
	High school, Science-Mathematics Program	144 (22.93)	19.80	26.39
Residential settings	Rural	289 (46.02)	42.15	49.94
	Peri-urban	158 (25.16)	21.91	28.71
	Urban	181 (28.82)	25.41	32.50
Raising animals (mammals)	No animals raised Livestock only	187 (29.78) 65 (10.35)	26.32 8.20	33.48 12.99
	Companion animals only	290 (46.18)	42.30	50.10
	Both livestock and companion animals	86 (13.69)	11.22	16.62
Have you studied on “zoonotic disease” before?	No/ Has not	339 (53.98)	50.06	57.85
	Yes/ Has	289 (46.02)	42.15	49.94
Has anyone in your family ever been infected with a zoonotic disease?	No/ Has not	609 (96.97)	95.30	98.06
	Yes/ Has	19 (3.03)	1.94	4.70
Have you ever been bitten by a dog?	No/ Has not	395 (62.90)	59.04	66.60
	Yes/ Has	233 (37.10)	33.40	40.96
Have you ever been bitten or scratched by a cat?	No/ Has not	368 (58.60)	54.69	62.40
	Yes/ Has	260 (41.40)	37.60	45.31
Have you ever been vaccinated against rabies?	No/ Has not	351 (55.89)	51.97	59.74
	Yes/ Has	277 (44.11)	40.26	48.03

Among them, about half of them (339, 53.98%) had not studied zoonotic diseases previously, and most of them (609, 96.97%) reported that no one in their families had any zoonotic infections before. More than half of them reported having been bitten by a dog (395, 62.90%) and a cat (368, 58.60%). They (351, 55.89%) also reported having received rabies vaccination, including both pre-exposure vaccination and post-exposure prophylaxis (PEP) following animal exposure (Table 1). Table 1 presents the sociodemographic characteristics of the secondary school study participants in Maha Sarakham Province, Thailand.

A comparison of respondents recruited through the on-site survey and the online questionnaire revealed no statistically significant differences in age, gender, or educational level (*p* > 0.05). Therefore, data from both collection methods were pooled for subsequent analyses.

### Knowledge, Attitude, Practices (KAP) scores for rabies prevention

The KAP scores for rabies prevention among the study participants revealed that the participants had a median knowledge score of 22/ 50, which was derived from the original 15-item knowledge questionnaire (IQR = 16), an attitude score with a median of 3.11 on a 5-point Likert scale (IQR = 0.51), and a practice score with a median of 28 (IQR = 10). All variables showed non-normal distributions.

For the KAP classifications, this study found that the majority of knowledge scores about rabies were at a moderate level (55.89%), followed by a poor level (33.28%) and a good level (10.83%), respectively. Most of them had neutral attitude scores toward rabies prevention (81.37%), followed by a positive attitude (10.67%) and a negative attitude (7.96%), respectively. As for the level of practices in rabies prevention, most of them scored at a moderate level (72.77%), followed by an unsatisfactory level (15.92%) and a good level (11.31%), respectively. The above analysis results of the KAP classifications are shown in Table 2.

**Table 2:** Knowledge, attitudes, and practices toward rabies prevention among students from secondary schools and vocational institutions in Maha Sarakham Province, Thailand (*n* = 628)

Items	Levels	Number (Percent)	Reliability (95%)	
			Lowest	Highest
Knowledge	Good	68 (10.83)	8.62	13.51
	Moderate	351 (55.89)	51.97	59.74
	Poor	209 (33.28)	29.70	37.07
Attitude	Positive	67 (10.67)	8.48	13.24
	Neutral	511 (81.37)	78.12	84.23
	Negative	50 (7.96)	6.08	10.36
Practice	Good	71 (11.31)	9.05	14.03
	Average	457 (72.77)	69.15	76.12
	Unsatisfactory	100 (15.92)	13.26	19.00

For comparative analysis of factors using the Shapiro-Wilk test for data distribution, this study found that the data distribution was non-normal. In this regard, the Mann-Whitney U test and Kruskal-Wallis’s rank test were used, and the results of the above comparative testing were shown in Table 3. The analysis revealed that participants who had experience raising mammalian pets had significantly higher knowledge scores regarding rabies prevention (*P-value* = 0.04). The participants who had previously studied “zoonotic diseases” had a significantly higher knowledge score than those who had not, with a *P-value* < 0.001. Additionally, the participants who had been bitten/ scratched by a cat before had a significantly higher knowledge score than those who had not, with a *P-value* of 0.01. Furthermore, the persons who had been vaccinated against rabies had a significantly higher knowledge score than those who had not been vaccinated, with a *P-value* < 0.001.

**Table 3:** Socio-demographic characteristics of students from secondary schools and vocational institutions in Maha Sarakham Province, Thailand by knowledge scores on rabies prevention, using the Mann-Whitney U test and Kruskal-Wallis’s rank test (*n* = 628)

Variables	Descriptions	Median	IQR	Range	*P*-value
Gender	Male	21.00	16.00	0–50	0.51
	Female	22.00	16.00	0–50	
	Others	26.50	15.50	0–50	
Age-groups	Under 15 years	20.00	17.00	0–50	0.79
	15 years of age	23.00	16.00	0–50	
	Over 15 years	21.00	16.00	0–50	
Educational attainment levels	Junior high school	22.00	17.00	0–50	0.32
	Vocational college	26.50	15.00	0–50	
	High school, Arts program, and other programs	21.00	13.00	0–50	
	High school, Science-Mathematics Program	22.00	17.50	0–50	
Residential settings	Rural	20.00	16.00	0–50	0.12
	Peri-urban	22.00	15.00	0–50	
	Urban	23.00	16.00	0–50	
Raising animals (mammals)	No animals raised Livestock only	20.00 21.00	15.00 13.00	0–50 0–50	0.04*
	Companion animals only	23.00	17.00	0–50	
	Both livestock and companion animals	23.00	15.00	0–50	
Have you studied on “zoonotic disease” before?	No/ Has not	19.00	16.00	0–50	0.00*
	Yes/ Has	24.00	14.00	0–50	
Has anyone in your family ever been infected with a zoonotic disease?	No/ Has not	21.00	16.00	0–50	0.14
	Yes/ Has	25.00	12.00	0–50	
Have you ever been bitten by a dog?	No/ Has not	21.00	17.00	0–50	0.12
	Yes/ Has	22.00	15.00	0–50	
Have you ever been bitten or scratched by a cat?	No/ Has not	21.0	16.00	0–50	0.01*
	Yes/ Has	23.00	16.00	0–50	
Have you ever been vaccinated against rabies?	No/ Has not	20.00	17.00	0–50	0.00*
	Yes/ Has	23.00	15.00	0–50	

* P-value < 0.05

In addition, Table 4 shows the comparative analysis results of rabies prevention knowledge among the study participants among animals, including companion and livestock animals, that were analyzed pairwise using the Mann-Whitney U test. The results showed that the scores on rabies prevention among the participants who did not keep the animals were significantly lower than those of the participants who kept the animals (*P-value* = 0.03) and participants who kept both companion animals and livestock (*P-value* = 0.03).

**Table 4:** Results of rabies prevention knowledge among the study participants in Maha Sarakham Province of Thailand by their experience in raising mammals, using the pairwise Mann-Whitney U test (*n* = 628)

Educational Attainment Levels	No animals raised	Livestock only	Companion animals only	Both livestock and companion animals
No animals raised	1.00			
Livestock only	0.83	1.00		
Companion animals only	0.03*	0.11	1.00	
Both livestock and companion animals	0.03*	0.07	0.49	1.00

* P-value < 0.05

Table 5 shows a comparative analysis of the basic factors of secondary school students with their attitudes toward rabies prevention, which was performed by testing the data distribution using the Shapiro-Wilk test, which revealed a non-normal distribution. Therefore, the Mann-Whitney U test and Kruskal-Wallis’s rank test were used. From the tests mentioned, the results showed that the differing educational backgrounds of the participants significantly affected their attitude scores toward rabies prevention, with a *P-value* < 0.001. Additionally, the participants who had previously studied “zoonotic diseases” had significantly higher attitude scores than those who had not, with a *P-value* of 0.02. In addition, a comparative analysis of the attitude scores on rabies prevention among the study participants was analyzed pairwise using the Mann-Whitney U test, is shown in Table 6.

**Table 5:** Results of socio-demographic information among the study participants in Maha Sarakham Province of Thailand by the attitude scores toward rabies prevention, using the Mann-Whitney U test and the Kruskal-Wallis’s rank test (*n* = 628)

Variables	Descriptions	Median	IQR	Range	*P*-value
Gender	Male	3.03	0.89	1–5	0.08
	Female	3.11	0.57	1–5	
	Others	3.11	0.51	1–5	
Age-groups	Under 15 years	3.14	0.65	1–5	0.18
	15 years of age	3.03	0.68	1–5	
	Over 15 years	3.14	0.62	1–5	
Educational attainment levels	Junior high school	3.03	0.68	1–5	0.00*
	Vocational college	3.45	0.85	1–5	
	High school, Arts program, and other programs	3.00	0.89	1–5	
	High school, Science-Mathematics Program	3.28	0.54	1–5	
Residential settings	Rural	3.03	0.57	1–5	0.58
	Peri-urban	3.07	0.73	1–5	
	Urban	3.14	0.62	1–5	
Raising animals (mammals)	No animals raised Livestock only	3.08 3.14	0.70 0.81	1–5 1–5	0.73
	Companion animals only	3.08	0.59	1–5	
	Both livestock and companion animals	3.03	0.57	1–5	
Have you studied on “zoonotic diseases” before?	No/ Has not	3.03	0.68	1–5	0.02*
	Yes/ Has	3.16	0.57	1–5	
Has anyone in your family ever been infected with a zoonotic disease?	No/ Has not	3.05	0.70	1–5	0.74
	Yes/ Has	3.19	0.51	1–5	
Have you ever been bitten by a dog?	No/ Has not	3.05	0.73	1–5	0.46
	Yes/ Has	3.11	0.68	1–5	
Have you ever been bitten or scratched by a cat?	No/ Has not Yes/ Has	3.05 3.11	0.73 0.50	1–5 1–5	0.98
Have you ever been vaccinated against rabies?	No/ Has not	3.05	0.81	1–5	0.61
	Yes/ Has	3.11	0.51	1–5	

* P-value < 0.05

**Table 6:** Results of the attitude scores on rabies prevention among the study participants in Maha Sarakham Province of Thailand, based on educational attainment levels, using the Mann-Whitney U test (*n* = 628)

Educational attainment levels	Junior high school	Vocational college	High school, Arts program, and other programs	High school, Science-Mathematics Program
Junior high school	1.00			
Vocational college	0.12	1.00		
High school, Arts program, and other programs	0.33	0.09	1.00	
High school, Science-Mathematics Program	0.00*	0.38	0.00*	1.00

* P-value < 0.05

In addition, the attitude scores toward rabies prevention among high school participants in the Science-Mathematics program were significantly higher than those of lower secondary school students (*P-value* < 0.001) and high school students in the Arts or other programs (*P-value* < 0.001).

Moreover, Table 7 shows the analysis results of the basic factors of high school and vocational participants to compare the performance scores supporting rabies prevention by testing the data distribution with the Shapiro-Wilk test, which found a non-normal distribution. Therefore, the Mann-Whitney U test and Kruskal-Wallis’s test rank test were performed. The results showed that gender was significantly associated with rabies preventive practice scores (*P-value* < 0.001), with female participants demonstrating significantly higher scores than male participants, consistent with the pairwise comparison analysis (Table 7). Age was also significantly associated with practice scores (*P-value* = 0.04). Additionally, it was found that the experience of raising pets in the mammal group significantly affected the practice scores with a *P-value* < 0.001.

**Table 7:** Results of the socio-demographic information among the study participants in Maha Sarakham Province of Thailand by their practice scores on rabies prevention, using the Mann-Whitney U test and Kruskal-Wallis’s rank test (*n* = 628)

Variables	Descriptions	Median	IQR	Range	*P*-value
Gender	Male	26.00	12.00	0–60	0.00*
	Female	30.00	11.00	0–60	
	Others	27.00	15.50	0–60	
Age-groups	Under 15 years	27.00	11.00	0–60	0.04*
	15 years of age	27.00	10.00	0–60	
	Over 15 years	30.00	12.00	0–60	
Educational attainment levels	Junior high school	27.00	11.00	0–60	0.08
	Vocational college	34.00	4.50	0–60	
	High school, Arts program, and other programs	30.50	13.00	0–60	
	High school, Science-Mathematics Program	30.00	12.00	0–60	
Residential settings	Rural	29.00	10.00	0–60	0.78
	Peri-urban	28.00	11.00	0–60	
	Urban	28.00	11.00	0–60	
Raising animals (mammals)	No animals raised Livestock only	27.00 26.00	12.00 11.00	0–60 0–60	0.00*
	Companion animals only	31.00	10.00	0–60	
	Both livestock and companion animals	30.00	11.00	0–60	
Have you studied on“zoonotic diseases” before?	No/ Has not	27.00	10.00	0–60	0.02*
	Yes/ Has	30.00	12.00	0–60	
Has anyone in your family ever been infected with a zoonotic disease?	No/ Has not	29.00	10.00	0–60	0.02*
	Yes/ Has	26.00	8.00	0–60	
Have you ever been bitten by a dog?	No/ Has not	28.00	10.00	0–60	0.67
	Yes/ Has	29.00	11.00	0–60	
Have you ever been bitten or scratched by a cat?	No/ Has not	27.00	11.00	0–60	0.01*
	Yes/ Has	30.50	9.50	0–60	
Have you ever been vaccinated against rabies?	No/ Has not	27.00	10.00	0–60	0.01*
	Yes/ Has	31.00	12.00	0–60	

* P-value < 0.05

Furthermore, the participants who had previously studied “zoonotic diseases” had significantly higher practice scores than those who had not studied “zoonotic diseases”, with a *P-value* of 0.02. However, it was found that participants with family members who had contracted zoonotic diseases had significantly lower practice scores than those whose family members had not contracted zoonotic diseases, with a *P-value* of 0.02. In terms of participants who had previously been bitten/ scratched by cats, they had significantly higher practice scores than those who had not been bitten/ scratched by cats, with a *P-value* of 0.01.

This study also revealed that the participants who had previously been vaccinated against rabies had significantly higher practice scores than those who had not been vaccinated, with a *P-value* of 0.01. The comparison of differences in gender, age, and experience in raising pets in the mammal group was analyzed pairwise using the Mann-Whitney U test.

In addition, the performance scores supporting rabies prevention among female participants were significantly higher than those of male students (*P-value* < 0.001). Pairwise comparisons further confirmed that female participants had significantly higher practice scores than male participants. In addition, participants aged over 15 years demonstrated significantly higher practice scores compared to younger age groups. Similarly, participants who raised companion animals or both companion animals and livestock had significantly higher practice scores compared to those who did not raise animals. It also revealed that the practice scores supporting rabies prevention among the participants without any animals were lower than those who kept animals (*P-value* < 0.001), and the persons with companion animals and livestock (*P-value* = 0.01) significantly.

### Bivariable analysis of factors associated with knowledge, attitudes, and practices

To identify factors associated with rabies-related knowledge, attitudes, and practices, participants were dichotomized into two groups based on the classifications presented in Table 2. Participants with good knowledge, positive attitudes, or good practices were compared with all remaining participants.

The bivariable analyses (Table 8) showed that educational level (*P* = 0.002), previous exposure to information on zoonotic diseases (*P* < 0.001), and a history of rabies vaccination (*P* = 0.007) were significantly associated with good knowledge. A history of cat scratches (*P* = 0.055) and previous zoonotic disease infection among participants or their family members (*P* = 0.140) also met the predefined criterion for inclusion in the multivariable analysis.

**Table 8:** Bivariable analysis of factors associated with good knowledge, positive attitude, and good practice regarding rabies prevention among participants (*n* = 628)

Variables	Knowledge		*P*-value	Attitude		*P*-value	Practice		*P*-value
	Good *n* = 68 (%)	Poor *n* = 560 (%)		Positive *n* = 67 (%)	Negative *n* = 561 (%)		Good *n* = 71 (%)	Poor *n* = 557 (%)	
Gender			0.569			0.203			0.014
Male	24 (35.3)	167 (29.8)		23 (34.3)	168 (29.9)		12 (16.9)	179 (32.1)	
Female	39 (57.4)	358 (63.9)		43 (64.2)	354 (63.1)		56 (78.9)	341 (61.2)	
Others	5 (7.4)	35 (6.2)		1 (1.5)	39 (7)		3 (4.2)	37 (6.6)	
Age-groups			0.647			0.544			0.034
Under 15 years	11 (16.2)	80 (14.3)		11 (16.4)	80 (14.3)		6 (8.5)	85 (15.3)	
15 years of age	29 (42.6)	216 (38.6)		22 (32.8)	223 (39.8)		22 (31)	223 (40)	
Over 15 years	28 (41.2)	264 (47.1)		34 (50.7)	258 (46)		43 (60.6)	249 (44.7)	
Educational attainment levels			0.002			0.068			0.113
Junior high school	41 (60.3)	313 (55.9)		35 (52.2)	319 (56.9)		31 (43.7)	323 (58)	
Vocational college	3 (4.4)	5 (0.9)		3 (4.5)	5 (0.9)		1 (1.4)	7 (1.3)	
High school, Arts program, and other programs	4 (5.9)	118 (21.1)		11 (16.4)	111 (19.8)		20 (28.2)	102 (18.3)	
High school, Science-Mathematics Program	20 (29.4)	124 (22.1)		18 (26.9)	126 (22.5)		19 (26.8)	125 (22.4)	
Residential settings			0.732			0.281			0.984
Rural	31 (45.6)	258 (46.1)		25 (37.3)	264 (47.1)		32 (45.1)	257 (46.1)	
Peri-urban	15 (22.1)	143 (25.5)		21 (31.3)	137 (24.4)		18 (25.4)	140 (25.1)	
Urban	22 (32.4)	159 (28.4)		21 (31.3)	160 (28.5)		21 (29.6)	160 (28.7)	
Raising animals (mammals)			0.364			0.069			0.285
No animals raised Livestock only	20 (29.4)	167 (29.8)		15 (22.4)	172 (30.7)		16 (22.5)	171 (30.7)	
Companion animals only	3 (4.4)	62 (11.1)		11 (16.4)	54 (9.6)		5 (7)	60 (10.8)	
Both livestock and companion animals	34 (50)	256 (45.7)		36 (53.7)	254 (45.3)		38 (53.5)	252 (45.2)	
No animals raised Livestock only	11 (16.2)	75 (13.4)		5 (7.5)	81 (14.4)		12 (16.9)	74 (13.3)	
Have you studied on“zoonotic diseases” before?			< 0.001			0.047			0.048
No/ Has not	20 (29.4)	319 (57)		28 (41.8)	311 (55.4)		30 (42.3)	309 (55.5)	
Yes/ Has	48 (70.6)	241 (43)		39 (58.2)	250 (44.6)		41 (57.7)	248 (44.5)	
Has anyone in your family ever been infected with a zoonotic disease?			0.140			1.000			1.000
No/ Has not	64 (94.1)	545 (97.3)		65 (97)	544 (97)		69 (97.2)	540 (96.9)	
Yes/ Has	4 (5.9)	15 (2.7)		2 (3)	17 (3)		2 (2.8)	17 (3.1)	
Have you ever been bitten by a dog?			0.546			0.330			0.574
No/ Has not	40 (58.8)	355 (63.4)		38 (56.7)	357 (63.6)		42 (59.2)	353 (63.4)	
Yes/ Has	28 (41.2)	205 (36.6)		29 (43.3)	204 (36.4)		29 (40.8)	204 (36.6)	
Have you ever been bitten or scratched by a cat?			0.055			0.266			0.427
No/ Has not	32 (47.1)	336 (60)		44 (65.7)	324 (57.8)		38 (53.5)	330 (59.2)	
Yes/ Has	36 (52.9)	224 (40)		23 (34.3)	237 (42.2)		33 (46.5)	227 (40.8)	
Have you ever been vaccinated against rabies?			0.007			0.593			0.068
No/ Has not	27 (39.7)	324 (57.9)		40 (59.7)	311 (55.4)		32 (45.1)	319 (57.3)	
Yes/ Has	41 (60.3)	236 (42.1)		27 (40.3)	250 (44.6)		39 (54.9)	238 (42.7)	

*P*-values were obtained using Pearson’s chi-square test or Fisher’s exact test, as appropriate

For attitudes, educational level (*P* = 0.068), ownership of mammalian companion animals (*P* = 0.069), and previous exposure to information on zoonotic diseases (*P* = 0.047) were associated with positive attitudes. Regarding practices, sex (*P* = 0.014), age (*P* = 0.034), educational level (*P* = 0.113), previous exposure to information on zoonotic diseases (*P* = 0.048), and a history of rabies vaccination (*P* = 0.068) were associated with good practices. Variables with *P* < 0.20 in the bivariable analyses were subsequently included in the corresponding multivariable logistic regression models.

### Factors associated with knowledge, attitudes, and practices: multivariable logistic regression analysis

The results of the multivariable logistic regression analyses are presented in Table 9. Variables with a *P*-value < 0.20 in the bivariable analyses were entered into the initial models, and backward stepwise elimination was applied to obtain the final models. For knowledge, previous exposure to information on zoonotic diseases (AOR = 2.98, 95% CI: 1.73–5.30; *P* < 0.001) and a history of rabies vaccination (AOR = 1.89, 95% CI: 1.12–3.24; *P* = 0.019) were independently associated with good knowledge. In contrast, students enrolled in arts and other academic programs were significantly less likely to have good knowledge than those in lower secondary education (AOR = 0.28, 95% CI: 0.08–0.72; *P* = 0.018). Although vocational students tended to have higher odds of good knowledge, this association was not statistically significant (AOR = 3.58, 95% CI: 0.68–16.18; *P* = 0.102).

**Table 9:** Multivariable logistic regression analysis of factors associated with good knowledge, positive attitude, and good practice regarding rabies prevention (*n* = 628)

Variables	ORs (95%CI)	crude *P*-value	AORs (95%CI)	*P* (Wald’s test)	*P* (LR-test)
Dependent; Good knowledge					
Educational attainment levels					0.005*
Junior high school (reference)	1		1		
Vocational college	4.58 (0.91, 19.38)	0.042*	3.58 (0.68, 16.18)	0.102	
High school, Arts program, and other programs	0.26 (0.08, 0.66)	0.011*	0.28 (0.08, 0.72)	0.018*	
High school, Science-Mathematics Program	1.23 (0.68, 2.16)	0.477	1.31 (0.72, 2.34)	0.366	
Have you studied on“zoonotic diseases” before?	3.18 (1.86, 5.60)	< 0.001*	2.98 (1.73, 5.3)	< 0.001*	< 0.001*
Have you ever been vaccinated against rabies?	2.08 (1.25, 3.52)	0.005*	1.89 (1.12, 3.24)	0.019*	0.018*
Dependent; Positive Attitude					
Educational attainment levels					0.201
Junior high school (reference)					
Vocational college	5.47 (1.09, 23.26)	0.024*	5.13 (1.01, 22.05)	0.031*	
High school, Arts program, and other programs	0.90 (0.42, 1.79)	0.779	0.93 (0.43, 1.84)	0.833	
High school, Science-Mathematics Program	1.30 (0.7, 2.36)	0.392	1.30 (0.70, 2.36)	0.397	
Have you studied on“zoonotic diseases” before?	1.73 (1.04, 2.92)	0.036*	1.69 (1.01, 2.86)	0.046*	0.044*
Dependent; Good practice					
Gender					0.010*
Male (reference)	1		1		
Female	2.45 (1.32, 4.90)	0.007*	2.51 (1.34, 5.08)	0.006*	
Others	1.21 (0.27, 4.04)	0.777	1.2 (0.26, 4.07)	0.788	
Age-groups					0.062
Under 15 years (reference)	1		1		
15 years of age	1.4 (0.58, 3.90)	0.484	1.28 (0.52, 3.61)	0.614	
Over 15 years	2.45 (1.08, 6.59)	0.049*	2.19 (0.95, 5.99)	0.089	
Have you studied on“zoonotic diseases” before?	1.7 (1.04, 2.83)	0.037*	1.71 (1.03, 2.87)	0.038*	0.037*
Have you ever been vaccinated against rabies?	1.63 (1.00, 2.70)	0.053	1.81 (1.09, 3.03)	0.023*	0.022*

Adjusted odds ratios (AORs) with 95% confidence intervals (CIs), *statistical significance at *P-value* < 0.05

For attitudes, vocational education (AOR = 5.13, 95% CI: 1.01–22.05; *P* = 0.031) and previous exposure to information on zoonotic diseases (AOR = 1.69, 95% CI: 1.01–2.86; *P* = 0.046) were independently associated with positive attitudes toward rabies prevention. For practices, female participants were more likely to demonstrate good rabies prevention practices than males (AOR = 2.51, 95% CI: 1.34–5.08; *P* = 0.006). Previous exposure to information on zoonotic diseases (AOR = 1.71, 95% CI: 1.03–2.87; *P* = 0.038) and a history of rabies vaccination (AOR = 1.81, 95% CI: 1.09–3.03; *P* = 0.023) were also independently associated with good practices. Older age showed a positive trend toward good practices but did not reach statistical significance (AOR = 2.19, 95% CI: 0.95–5.99; *P* = 0.089).

### Associations of Knowledge, Attitude, Practices (KAP) scores with the rabies prevention practices among secondary school students/ participants

**Fig. 2:**
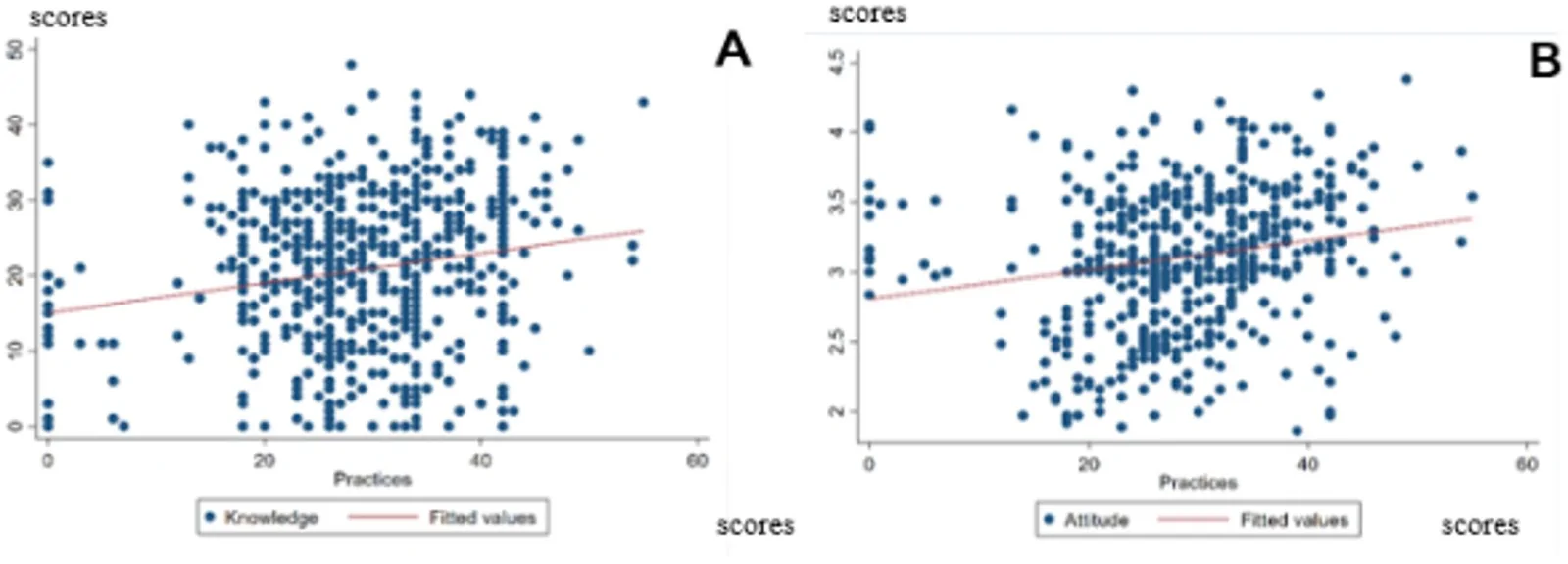
**A** presents a scatter plot illustrating the positive correlation between knowledge scores and practice scores in rabies prevention. **B** presents a scatter plot illustrating the positive correlation between attitude scores and practice scores in rabies prevention

The strength and direction of the associations among knowledge, attitude, and practice (KAP) scores were assessed using Spearman’s rank correlation coefficient (rₛ). Spearman’s correlation analysis revealed that both knowledge and attitude scores were positively associated with rabies preventive practice scores, with correlation coefficients of rₛ = 0.14 and rₛ = 0.28, respectively (*P* < 0.001). These findings indicated weak and weak-to-moderate positive correlations between the KAP domains. Attitude also has a positive correlation with rabies prevention practices at a low to moderate level (rₛ = 0.28) and is statistically significant (*P-value* < 0.001). The obtained data can be presented in a correlation graph as shown in Fig. 2. The practice of rabies prevention in form B shows a scatter plot graph illustrating the positive correlation between attitude scores and practice scores in rabies prevention. Detailed pairwise comparison results and additional statistical analyses are provided in the Supplementary Materials.

## Conclusion and discussion

This current study determined the KAP of high school and vocational students in Maha Sarakham province of Thailand regarding rabies prevention. The findings revealed that the participants’ knowledge levels were predominantly moderate, with a smaller proportion demonstrating high knowledge. Attitudes were largely neutral, and preventive practices were moderate, primarily reflecting gaps in both awareness and behavior. The present results were aligned with the previous study in Chonburi province of Thailand in 2000–2001 that among 290 primary school students, 52.1% had poor knowledge, had favorable attitudes (89.0%), and exercised proper preventive behaviors (52.1%) [16]. While in South Bhutan, which has rabies as an endemic disease, a study showed that among 701 school students, most of them demonstrated a good level of knowledge (59.7%) and a favorable perception towards rabies (57.7%) [13]. The higher level of rabies knowledge reported in Bhutan compared with the present study may be attributed to the endemic nature of rabies in the study areas, where frequent rabies outbreaks, government-led awareness campaigns, and annual educational activities on dog-bite prevention and rabies conducted by animal and public health authorities during World Rabies Day may have increased public exposure to rabies-related information [13].

The current study also identified several sociodemographic and previous experiences associated with higher KAP scores. For example, those who had prior experience of cat bites or scratches, or vaccination against rabies, or formal education on zoonotic diseases demonstrated significantly higher knowledge and practice scores. Similarly, the participants in science-related educational tracks and those with prior exposure to zoonotic disease education exhibited more positive attitudes toward rabies prevention. The aforementioned findings might be consistent with the Bhutan study [13], suggesting that prior experiences or information regarding rabies, as well as communication campaigns, could enhance KAP scores.

Gender and age significantly influenced the current findings, as female and older participants exhibited greater levels of preventive practices. The results contrast with a study conducted in Nepal, which indicated that male participants of younger age, possessing higher education and social status, exhibited greater knowledge regarding rabies [24].

The correlation analysis results further highlighted that both knowledge and attitudes were positively associated with preventive practices, though only at low to moderate levels. This suggests that while improving knowledge and shaping positive attitudes are necessary, they might not be sufficient alone to ensure high compliance with preventive practices. These results are consistent with prior KAP studies in rabies prevention among youth and communities in endemic regions, which emphasize the interplay of knowledge, experiential factors, and socio-cultural influences in shaping preventive behaviors [25, 26].

Although several findings in this study may align with expected patterns, the results provide important context-specific insights into rabies-related knowledge, attitudes, and practices among adolescents in a rabies-endemic setting. Notably, despite prior exposure to rabies-related experiences, including animal bites and vaccination, most participants demonstrated only moderate levels of knowledge and preventive practices. This suggests that exposure alone does not necessarily translate into effective understanding or behavior. Furthermore, the observed weak to moderate correlations between knowledge, attitudes, and practices indicate that improving awareness may not be sufficient to ensure appropriate preventive actions. These findings highlight the need for more comprehensive, behavior-focused interventions. In addition, the identification of disparities in preventive practices among male and younger students provides important direction for targeted public health strategies. Together, these findings contribute novel insights into the gap between awareness and behavioral outcomes in rabies prevention among adolescents.

### Recommendations

From a public health perspective, the findings suggest that rabies prevention among adolescents requires comprehensive, school-based strategies that extend beyond knowledge dissemination. Integrating rabies and zoonotic disease education into school curricula across disciplines can equip students with critical health literacy, ensuring equitable access to preventive knowledge [27]. Experiential approaches—such as school-based vaccination campaigns, interactive workshops, and collaboration with local health units—would reinforce behavior change and foster trust in health interventions [28, 29]. Tailored strategies for younger and male students, who exhibited weaker practices, should be prioritized, using peer-to-peer education, digital learning tools, and gender-sensitive communication [30]. These measures would directly strengthen community preparedness, reduce risky interactions with animals, and ultimately decrease human exposures to rabies.

From a One Health perspective, the findings of this study highlight the interconnected roles of human behavior, animal health, and public awareness in rabies prevention. The observed variations in rabies-related knowledge, attitudes, and practices among students suggest that educational and community-based factors may influence disease prevention behaviors. These findings support previous evidence emphasizing the importance of integrating human and animal health considerations in rabies control programs [30]. Furthermore, improving rabies awareness among school-aged populations may contribute to broader rabies prevention efforts and support the goals of the global “Zero by 30” initiative to eliminate human deaths from dog-mediated rabies by 2030 [30]. Future research should further investigate factors associated with rabies-related behaviors and evaluate the effectiveness of educational interventions within a One Health framework [31].

Finally, the study highlights the value of adolescent engagement as a public health resource. High school and vocational students can act as agents of change by transmitting knowledge and responsible practices to their families and communities, amplifying the reach of rabies prevention programs [30]. Empowering youth through education, vaccination access, and One Health–based activities not only supports rabies control but also contributes to broader zoonotic disease preparedness and resilience.

Beyond rabies, the strengthening of zoonotic literacy, intersectoral coordination, and community-based prevention demonstrated in this study provides a transferable model for wider pandemic preparedness. Early awareness and responsible behaviors in school-aged populations create a foundation for societal resilience against emerging infectious diseases [31]. By embedding rabies prevention within a One Health framework, Thailand not only advances progress toward rabies elimination but also builds the social, educational, and health system capacities required to detect, prevent, and respond to future zoonotic threats.

### Limitations

This study has several limitations. First, recall bias might have occurred, as participants were asked to report previous experiences with animals, which they might not accurately remember. Second, both face-to-face interviews and online questionnaires were used for data collection. Although no significant differences in sociodemographic characteristics were observed between these groups, the use of mixed methods may have introduced selection or response bias. Third, the total number of students invited to participate was not recorded, therefore, response and completion rates could not be calculated, limiting the assessment of potential non-response bias. Additionally, only fully completed questionnaires were included in the analysis, which may have affected the representativeness of the findings.

## Supplementary Information

Supplementary Material 1.

## Data Availability

The datasets generated and analyzed during the current study are available from the corresponding author upon reasonable request.
