# Supplementary material for: Determinants of rabies knowledge, attitudes, and practices among secondary and vocational school students in Maha Sarakham Province, Thailand
Source: BMC Vet Res. 2026 Aug 3;22:646. doi: 10.1186/s12917-026-05796-y (PMC13726079; doi:10.1186/s12917-026-05796-y)
Supplement: Supplementary file 1 [file 12917_2026_5796_MOESM1_ESM.docx]

**Supplement Table 1:** The question topics on the aspects of knowledge, attitude, and practices (KAP) related to rabies^[1]^

| **Sections** | **Measurement scales** | **Aspects** | **Topic of the question** | **Item Score** |
| --- | --- | --- | --- | --- |
| 1) Knowledge  (15 items) | Response format: True / False  Item Scoring: Correct = 1 point; Incorrect = 0 points Total item score range: 0–15 and convert to 50-point scale.  Classification: Poor (0–16.66), Fair/Moderate (16.67–33.33), Good (33.34–50) | 1.1) The basics of the disease | 1. Rabies is a viral disease that affects the nervous system, leading to acute inflammation of the brain. 2. The cause of rabies is a virus 3. Rabies occurs at any time and in anytime/any season 4. Rabies can infect all mammals 5. Humans can be affected by rabies 6. Major reservoirs are free-roaming and unvaccinated dogs and cats 7. Rabies can be transmitted from animals to animals 8. Rabies can be transmitted from pets to humans 9. Rabies can be transmitted from livestock to humans   10. The main mode of transmission is bitten from dogs and scratched from cats  11. Two clinical forms of rabies are the furious and paralytic forms  12. Rabies is a fatal infectious disease; it cannot be treated after the onset of clinical signs | 1 / 0  1 / 0  1 / 0  1 / 0  1 / 0  1 / 0  1 / 0  1 / 0  1 / 0  1 / 0  1 / 0  1 / 0 |
|  |  | 1.2) Key to preventive information | 13. Rabies infection can be prevented and controlled by vaccination  14. Pets were required to boost rabies vaccines annually  15. Post-exposure prophylaxis can prevent severe cases of human and mortality | 1 / 0  1 / 0  1 / 0 |
| 2) Attitude  (10 items) | Response format: 5 point Likert scale (1 = Strongly disagree, 2 = Disagree, 3 = Neutral, 4 = Agree, 5 = Strongly agree) Scoring: 1–5 per item Total item score range: 10–50.  The total attitude item score was converted to a 5-point scale.  Classification: Negative (1.00–2.33), Neutral (2.34–3.67), and Positive (3.68–5.00) | 2.1) Students’ opinions of the severity of rabies infection | 16. You consider that rabies is a public health problem in many countries all over the world.  17. You consider that you and your family are at risk of rabies infection  18. You consider that the eradication of rabies is vital | 1–5  1–5  1–5 |
|  |  | 2.2) Wrong attitude about rabies among the students | 19. You think that the meat or liver consumption of a rabid animal could be a medication for rabies  20. You think that using shoes to slap the dog bite wounds can prevent rabies infection. | 1–5  1–5 |
|  |  | 2.3) Students’ perspectives on preventive approaches | 21. You consider that the key components in the prevention of rabies are both humans and animals  22. You think that pet owners should be responsible for rabies prevention  23. If you are a pet owner, you need to take your pets for rabies vaccination annually.  24. If you get bitten by an animal, you will go to take Rabies post-exposure prophylaxis (PEP)  25. You consider that the education of zoonosis, including rabies, is necessary for students. | 1–5  1–5  1–5  1–5  1–5 |
| 3) Practice  (15 items) | Response format: Yes / Sometimes / No Scoring: Yes = 2, Sometimes = 1, No = 0 Total Practice item score range: 0–30.  The total Practice item score was converted to a to a 60-point scale.  Classification: Unsatisfactory (0.00–20.00), Average (21.00–40.00), or Good (41.00–60.00) practice. | 3.1) Students’ behavior that involves the risk of rabies infection | 26. You have participated in an education or vaccination campaign or workshop about rabies  27. You had received the rabies vaccine (PrEP or PEP) before or after you were bitten by dogs or scratched by cats  28. You don't tease dogs or cats while they are eating food. | 2 / 1 / 0  2 / 1 / 0  2 / 1 / 0 |
|  |  | 3.2) Students’ practices of preventive approaches | 29. Have you ever captured and observed a suspected rabid animal, or reported it for observation? 30. When you see a suspected rabid animal, do you contact officers from the Department of Livestock Development? 31. After being bitten or scratched by a suspected animal, do you wash the wound with water and soap? 32. After washing the wound, do you go to a healthcare center or hospital for treatment? 33. Do you report animal bite incidents to both public health and veterinary authorities? 34. After a suspected animal bite, do you receive anti-rabies vaccination immediately? 35. Do you have your pets vaccinated against rabies by a licensed veterinarian? 36. Do you vaccinate puppies against rabies before three months of age (if applicable)? 37. Do you keep your pets in a secure area, such as a cage or within a fenced property? 38. Do you regularly observe and monitor changes in your pets’ behavior? 39. Do you prevent your pets from contacting wild animals? 40. Do you wash your hands after contacting animals? | 2 / 1 / 0  2 / 1 / 0  2 / 1 / 0  2 / 1 / 0  2 / 1 / 0  2 / 1 / 0  2 / 1 / 0  2 / 1 / 0  2 / 1 / 0  2 / 1 / 0  2 / 1 / 0  2 / 1 / 0 |

# **Supplement Table 2.** Distribution of knowledge, attitude, and practice scores for rabies prevention among secondary school study participants in Maha Sarakham Province, Thailand (n = 628)

| **Items** | **Mean ± SD** | **Median** | **IQR** | **Max** | **Min** | **Distribution** |
| --- | --- | --- | --- | --- | --- | --- |
| Knowledge (50 points) | 20.71 ± 10.69 | 22 | 16 | 48 | 0 | Non-normal |
| Attitude (5 points) | 3.11 ± 0.51 | 3.08 | 0.7 | 4.37 | 1.86 | Non-normal |
| Practice (60 points) | 28.61 ± 9.65 | 28 | 10 | 55 | 0 | Non-normal |

# **Supplement Table 3.** Results of rabies prevention practice scores among the study participants in Maha Sarakham Province of Thailand by gender, using the pairwise Mann-Whitney U test (n = 628)

| **Gender** | **Male** | **Female** | **Others** |
| --- | --- | --- | --- |
| Male | 1.00 |  |  |
| Female | 0.00* | 1.00 |  |
| Others | 0.87 | 0.08 | 1.00 |

** P-value < 0.05*

# **Supplement Table 4.** Results of rabies prevention practice scores among the study participants in Maha Sarakham Province of Thailand by age-groups, using the pairwise Mann-Whitney U test (n = 628)

| **Age-groups** | **Under 15 years** | **15 years of age** | **Over 15 years** |
| --- | --- | --- | --- |
| Under 15 years | 1.00 |  |  |
| 15 years of age | 0.24 | 1.00 |  |
| Over 15 years | 0.02* | 0.09 | 1.00 |

** P-value < 0.05*

# **Supplement Table 5.** Results of rabies prevention practice scores among the study participants in Maha Sarakham Province of Thailand by mammal raising experiences, using the pairwise Mann-Whitney U test (n = 628)

| **Mammal raising experiences** | **No raising of the livestock** | **Livestock is raised** | **Having domestic animals** | **Having livestock and domestic animals** | |
| --- | --- | --- | --- | --- | --- |
| No raising of the livestock | 1.00 |  |  | |  |
| Livestock is raised | 0.47 | 1.00 |  | |  |
| Having domestic animals | 0.00* | 0.15 | 1.00 | |  |
| Having livestock and domestic animals | 0.01* | 0.10 | 0.62 | | 1.00 |

** P-value < 0.05*

# **Supplement Table 6.** Spearman's correlation coefficient analysis results regarding the relationship between knowledge, attitude, and practice scores on rabies prevention among secondary school study participants in Maha Sarakham Province, Thailand (n = 628)

| **r (P-value)** | **Knowledge** | **Attitude** | **Practice** |
| --- | --- | --- | --- |
| Knowledge | 1.00 |  |  |
| Attitude | 0.07 (0.07) | 1.00 |  |
| Practice | 0.14 (0.00*) | 0.28 (0.00*) | 1.00 |

** P-value < 0.05*

1. *This questionnaire was adapted from the contents and formats of a previous study carried out by Kiratitana-Olan et al. (2021), titled Keys to Good Knowledge, Attitude and Practice for Rabies Prevention in Disease-Free Communities in Thailand.* *Veterinary Integrative Sciences, 19(3), 407-422.*
